# Manual Dexterity Training and Cognitive Function in Adults with Stroke: A Scoping Review

**DOI:** 10.3390/healthcare14020234

**Published:** 2026-01-17

**Authors:** Gema Moreno-Morente, Verónica Company-Devesa, Cristina Espinosa-Sempere, Paula Peral-Gómez, Vanesa Carrión-Téllez, Laura-María Compañ-Gabucio

**Affiliations:** 1 Departamento de Patología y Cirugía, Universidad Miguel Hernández (UMH), 03550 Alicante, Spain; veronica.company@umh.es (V.C.-D.); c.espinosa@umh.es (C.E.-S.); pperal@umh.es (P.P.-G.); vcarrion@umh.es (V.C.-T.); lcompan@umh.es (L.-M.C.-G.); 2 Being + Doing & Becoming Occupational Research Group (B+D+b), Miguel Hernández University, 03550 Alicante, Spain; 3 Instituto de Investigación Sanitaria y Biomédica de Alicante (ISABIAL), 03010 Alicante, Spain; 4 Unidad de Epidemiología de la Nutrición, Departamento de Salud Pública, Historia de la Ciencia y Ginecología, Universidad Miguel Hernández (UMH), 03550 Alicante, Spain; 5 CIBER Epidemiología y Salud Pública (CIBERESP), Instituto de Salud Carlos III, 28029 Madrid, Spain

**Keywords:** stroke, acquired brain injury, cognitive, manual dexterity, upper limb, occupational therapy, review

## Abstract

**Background:** Acquired brain injury (ABI) affects manual dexterity (MD) and cognitive functions, limiting daily activity performance. Occupational therapy aims to improve functionality and quality of life. **Objective:** To examine and describe the available evidence on the impact of MD training on cognitive processes and functional performance in adults with stroke, as well as to identify the most commonly used assessment tools and intervention techniques. **Methods:** Scoping review. A systematic literature search was conducted in PubMed and Scopus to identify experimental studies from the last 10 years involving adults with ABI who participated in interventions targeting upper-limb, MD, and cognitive function. A three-phase screening was carried out by two authors with duplicates removed using Zotero version 7.0. **Results:** Ten articles published between 2016 and 2023 were included. The most frequent interventions involved robotics and virtual reality. Eight studies were conducted by occupational therapists or included occupational therapy involvement, while two were conducted by physiotherapists. Training MD and upper-limb motor skills led to improvements in attention, memory, and executive functions. **Conclusions:** Findings support combined motor–cognitive interventions carried out by occupational therapists or physiotherapists to optimize rehabilitation outcomes, although further research is needed to strengthen the evidence.

## 1. Introduction

Acquired brain injury (ABI) is defined as a sudden-onset, non-degenerative brain injury affecting a previously healthy and fully developed brain [1]. This condition is highly heterogeneous, as its sequelae vary according to the location, extent, and severity of the lesion [2]. ABI can result from several causes, including cerebrovascular accidents (stroke), traumatic brain injury (TBI), anoxic or hypoxic episodes, and central nervous system tumors or infections [3]. Among these, stroke is the most frequent cause of ABI, followed by TBI, while other etiologies are less common [4].

Stroke is the second leading cause of death and the leading cause of disability worldwide, with a global age-standardized prevalence exceeding 1200 cases per 100,000 population [5]. More than 30% of survivors experience sequelae leading to varying degrees of disability or dependence, making stroke the leading cause of dependence in the country [6]. The sequelae of stroke are multiple and heterogeneous, including motor, sensory, cognitive, emotional, language, and swallowing impairments [7]. These deficits directly affect personal autonomy and performance in activities of daily living (ADLs), thereby hindering the ability to perform dual tasks that require cognitive functions, such as talking while dressing [8]. A reduced capacity to perform dual tasks can be detrimental to independence in daily life [9].

Cognitive processes, such as memory, attention, orientation, and decision-making, are essential for effective interaction with the environment [9]. At the same time, sensorimotor impairments of the UL, such as hemiparesis, loss of fine motor coordination, spasticity, and sensory deficits, are common after stroke [10,11], particularly affecting manual dexterity (MD) [12,13] and compromising object manipulation and task performance [14]. In this sense, MD refers to precise, coordinated hand and finger movements during goal-directed tasks, UL motor function encompasses motor control, strength, and coordination of the shoulder, arm, and hand, and fine motor skills are considered a subset of distal UL movements involving small, precise actions.

Since stroke can cause combined physical, cognitive, and emotional impairments, functional outcomes vary widely among individuals, ranging from full recovery to severe dependence or coma [4]. Among the disciplines involved in ABI rehabilitation, occupational therapy (OT) plays a key role in promoting maximal autonomy and functional independence [15]. OT employs a variety of strategies to improve MD, including upper-limb (UL) training, the use of assistive devices, fine motor exercises, bimanual coordination training, virtual reality (VR), and sensory stimulation, among others [14]. All interventions are individualized according to the clinical needs and characteristics of each person.

From a neuroscientific perspective, the concepts of brain connectivity—which highlights the functional interrelation between different brain regions [16]—and neuroplasticity—the ability of the nervous system to reorganize in response to experience or learning [17]—suggest that simultaneous activation of motor and sensory areas during MD training may promote the reorganization of neural networks. This process could positively influence cognitive functions such as attention, working memory, and executive functions, which are directly associated with motor learning after stroke, indicating that motor recovery cannot be understood in isolation from underlying cognitive processes.

In this regard, theoretical frameworks such as embodied cognition, which links action and cognition [18]; the cognitive-motor interference theory, which describes the competition for shared resources between motor and cognitive tasks [8]; and principles of task-oriented motor learning, which emphasize the importance of meaningful, task-specific practice [19], provide a solid conceptual foundation for understanding the interaction between MD and cognitive functions in post-stroke rehabilitation.

Furthermore, these theories are supported by recent evidence demonstrating a close relationship between MD and cognitive processes. Studies in older adults have shown that reduced MD is associated with cognitive impairments, particularly in executive functions, with significant correlations between performance on the Trail Making Test and dexterity tasks such as finger tapping [20]. Similar associations have been observed in individuals with Parkinson’s disease, where fine motor skills correlate with visual search abilities and motor speed, whereas gross motor skills show inverse associations with cognitive abilities [21]. Additionally, research in both younger and older adults indicates that executive functions are a significant predictor of movement times and motor accuracy, reinforcing the strong link between higher-level cognitive processes and manual performance [22].

Understanding the cognitive mechanisms that contribute to the recovery of MD after stroke is essential to explain functional deficits in daily life, guide study design, support evidence-based clinical decision-making, and develop neurorehabilitation programs aimed at optimizing functional recovery. Therefore, the primary objective of this scoping review is to examine and describe the available evidence on the impact of MD training on cognitive processes and functional performance in adults with stroke, as well as to identify the most commonly used assessment tools and intervention techniques.

## 2. Materials and Methods

A scoping review was systematically conducted in accordance with the Cochrane Handbook for Systematic Reviews [23] and the PRISMA Extension for Scoping Reviews (PRISMA-ScR) guidelines [24] (Table S1). A scoping review approach was chosen because it is specifically designed to address broad research questions, allowing researchers to map existing evidence, clarify conceptual boundaries, and identify gaps in knowledge, rather than evaluate intervention effectiveness [25,26]. The review procedure followed the five methodological stages described by Arksey and O’Malley [27,28] and enhanced by Levac et al. [29]: identifying the research question; identifying relevant studies; selecting studies through independent screening; charting the data using a standardized extraction form; and summarizing and reporting the results in a narrative and descriptive manner. The review protocol was registered in the Open Science Framework https://osf.io/bt354/overview (accessed on 13 December 2025).

### 2.1. Search Strategy

In January 2025, two databases were consulted: PubMed and Scopus. The same search terms were applied across all databases, combined using the Boolean operator AND. In Scopus, additional filters were applied within the “Subject Area” categories [Neuroscience/Health Professions/Psychology]. These filters were necessary, as without them the search yielded an excessively large number of results, most of which were unrelated to MD, given the broad use of the term “rehabilitation”. Only studies published in the past 10 years were included in order to provide a current synthesis of information. The time frame for considering an article to be current or not varies between disciplines, although in health and medical sciences, it is recommended to use references from the last 5 years [30]. However, we opted for a 10-year window to maximize coverage without including outdated interventions. All search strategies are shown in Table 1.

### 2.2. Eligibility Criteria

Specifically, articles were required to meet the following criteria to be included in this review:

- Studies published in English or Spanish.
- Studies with full text available.
- Experimental study designs, including randomized controlled trials, non-randomized controlled trials, pilot and exploratory experimental studies.
- Studies involving adults aged 18 years or older with any type of ABI.
- Studies aimed at improving UL motor skills or MD.
- Studies addressing cognitive processes, either directly or indirectly.

Articles were excluded if they met the following criteria:

- Studies published in other languages.
- Studies whose intervention was not focused on MD.
- Studies with the following designs: abstracts, editorials, letters to the editor, opinions, reviews, brief reports, conference papers, books, book chapters, scale validation studies, qualitative studies, case reports, animal studies, case series studies, observational studies, and protocols.

All inclusion and exclusion criteria were applied manually.

### 2.3. Study Selection

The titles of articles retrieved from the two databases were exported into a Microsoft Excel spreadsheet. A preliminary removal of duplicates was conducted, followed by a two-stage screening process: first, through title and abstract review, and subsequently, via full-text evaluation. An exhaustive review was carried out to select articles that met the inclusion criteria, discarding those that did not. The Excel database was created and duplicates removed by one researcher (GMM), while two researchers (VCD and LMCG) independently screened the articles. In cases of disagreement, the inclusion criteria were reviewed and discussed jointly; if consensus could not be reached, a third researcher (PPG) acted as an arbitrator, applying the predefined eligibility criteria to make the final decision.

### 2.4. Data Extraction and Synthesis

Prior to data extraction, a table was created to compile information from the articles, aiming to facilitate a more objective data synthesis and to prevent data manipulation. The table, “General Characteristics of Included Studies”, included the PICOS parameters (Population, Intervention, Comparator, Outcome, and Study Design) for each study: author/year, participants, intervention/comparator, study outcome, and assessment tool. Data extraction was performed independently by two authors (GMM and VCD) using a standardized form. Disagreements were resolved through discussion or consultation with a third author (LMCG).

### 2.5. Quality Assessment

As recommended for scoping reviews, no formal risk of bias assessment was conducted. In addition, the methodological quality of the included studies was not assessed, as this step is not a mandatory component of scoping reviews [27,28,29]. The primary purpose of a scoping review is to map the existing evidence and describe the breadth and nature of the available literature rather than to evaluate methodological rigor.

## 3. Results

**Figure 1:**
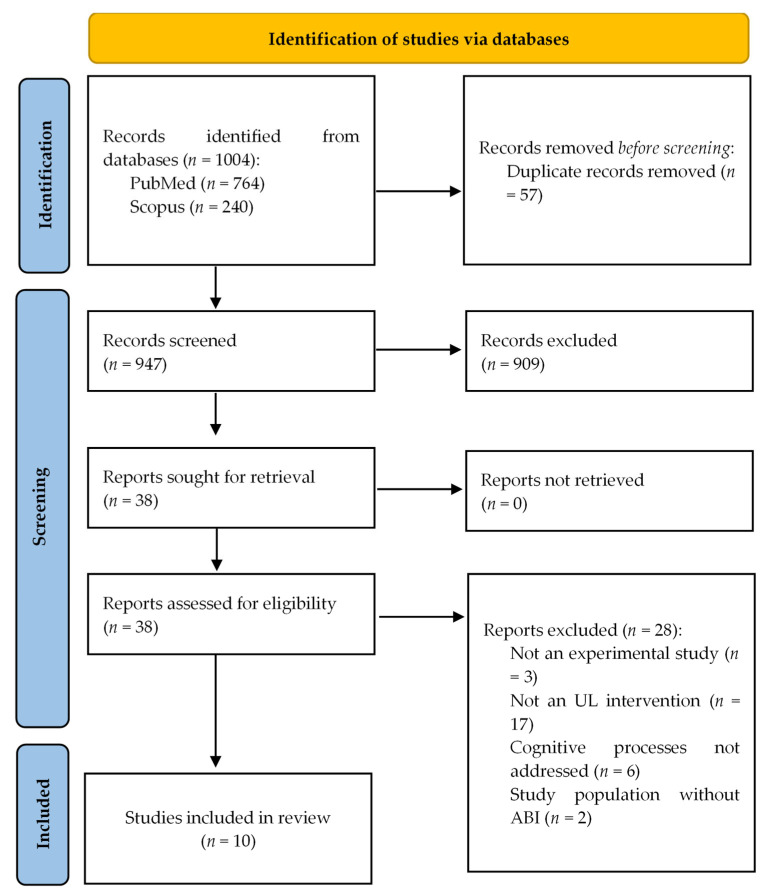
Flowchart of the study selection.

A total of 1004 articles were retrieved from the bibliographic searches conducted in the two consulted databases. After removing duplicates, 947 articles remained for title and abstract screening. Of these, 38 articles were selected for full-text review, and ultimately, 10 articles that met the inclusion criteria were included in this scoping review (Figure 1).

### 3.1. Main Characteristics of the Included Studies

The studies were conducted in the Republic of Korea (*n* = 3), Turkey (*n* = 2), Australia (*n* = 2), Italy (*n* = 1), the United States (*n* = 1), and China (*n* = 1). All included studies were randomized clinical trials, with only one pilot study identified [31].

### 3.2. Study Population in the Included Studies

Across the included studies, participants’ ages ranged from 53 to 73 years. There was notable variability in sample sizes, which ranged from 17 to 60 participants, with most studies recruiting between 30 and 40 individuals. Regarding gender distribution, all studies included male and female participants. A predominance of male participants was observed in six studies [32,33,34,35,36,37], whereas female predominance was reported in three studies [31,38,39]. One study [40] did not explicitly report sex distribution data. The disease duration also varied substantially among studies, ranging from a minimum reported duration of 18.25 days to an average duration of 33.72 months in the study conducted by Ersoy et al. (2020) [32]. All included studies investigated the same pathology, stroke.

### 3.3. Main Intervention Characteristics of the Included Studies

In all cases, different interventions were carried out in the control and intervention groups (*n* = 10), with the following comparisons standing out: real boxing vs. virtual boxing [32], hand training with the Amadeo™ robotic device (Tyromotion, Marietta, GA, USA) and VR vs. hand training without the device and VR [38]; hand training with VR and real objects vs. conventional OT [33]; robotic rehabilitation vs. conventional OT [34]; VR with the RAPAEL glove vs. recreational activities and conventional treatment [35]; conventional treatment vs. conventional treatment plus Motor imagery training with brain–computer interface (MI-BCI) [36]; dual-task training vs. conventional treatment [40]; Elements VR vs. conventional treatment [31]; virtual rehabilitation with EDNA-22 plus conventional treatment vs. the GRASP UL training program [37]; and finally, Cognitive Orientation to daily Occupational Performance (CO-OP) combined with task-oriented practice vs. task-oriented practice alone [39].

Regarding the objectives of the interventions in the selected studies, only one focused specifically on hand function and MD [38]. Five studies addressed UL function more broadly, incorporating MD as part of a global UL rehabilitation approach [32,33,34,37,39], while in four studies, MD was not explicitly specified as an intervention objective, and outcomes were reported for UL function in general [31,35,36,40]. Of the 10 included studies, 8 involved occupational therapists directly in the interventions [31,33,35,36,37,38,39,40], and 2 were conducted by physiotherapists [32,34].

### 3.4. Variables of Study and Measurement Instruments

A wide range of outcome variables was assessed across the included studies. These variables covered motor, cognitive, functional, emotional, and quality-of-life domains.

#### 3.4.1. Motor and Sensorimotor Function

Variables evaluated included UL motor function, DM, muscle strength, spasticity, and balance. Instruments used were:

Wolf Motor Function Test (WMFT) [32,35,36], Fugl-Meyer Assessment Upper extremity (FMA-UE) [33,34,36,38,40], Box and Block Test (BBT) [31,33,35,37], Nine Hole Peg Test (9HPT) [33,37], Jebsen-Taylor Hand Function Test (JTHFT) [35], Action Research Arm Test (ARAT) [39], Minnesota Manual Dexterity Test (MMDT) [32,34], Purdue Pegboard [34], Manual Muscle Test [33], Handgrip Test [33,34,35], Modified Ashworth Scale (MAS) [33,34], Berg Balance Scale [40], Fullerton Advanced Balance Test [32], and Modified Functional Reach Test [40].

#### 3.4.2. Cognitive Function

Cognitive domains assessed included global cognition, executive function, memory, attention, constructive praxis, and processing speed. Instruments used were:

Addenbrooke’s Cognitive Examination-Revised (ACE-R) [32], Montreal Cognitive Assessment (MoCA) [31,33,34,37,38], Mini-Mental State Examination (MMSE) [33], Frontal Assessment Battery (FAB) [38], Attentive Matrices (AM) [38], Digit Span (DS) [38,40], Rey–Osterrieth complex figure (ROCF) [38], Weigl Test [38], Trail Making Test (TMT) [35,39,40], Attention Network Test (ANT) [36], Schulte Grid Test (SGT) [36], Symbol Digit Modalities Test (SDMT) [36], Stroop Color & Word Test [40], Groton Maze Learning Task (GMLT) [31], Set-Shift Task (SST) [31], and Delis–Kaplan Executive Function System (D-KEFS) [39].

#### 3.4.3. Functional Performance and Daily Living

Functional independence, occupational performance, and ADLs were assessed using: Functional Independence Measure (FIM) [34], Modified Barthel Index [36], Nottingham Extended ADL [34], Canadian Occupational Performance Measure (COPM) [39], and Performance Quality Rating Scale [39].

#### 3.4.4. Quality of Life and Psychological Variables

Quality of life and emotional status, including depression, were measured using: Stroke-Specific Quality of Life Scale (SS-QOL) [34], Stroke Impact Scale [37,39], and Center for Epidemiologic Studies Depression Scale (CES-D) [34].

### 3.5. Occupational Therapy Interventions

**Figure 2:**
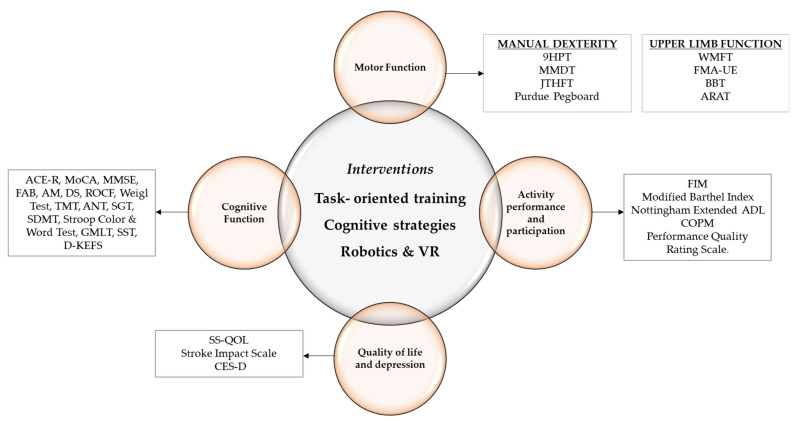
Conceptual overview of the interventions, outcomes, and assessment tools reported in the included studies. The central circle depicts the interventions implemented in the reviewed studies, the outer circles represent the assessed outcomes, and the squares correspond to the assessment tools used to evaluate these outcomes.

The characteristics of the interventions used in the selected studies are described below (Table 2). Given the heterogeneity of the intervention approaches and outcome domains identified across the included studies, an integrative conceptual mapping was developed to support interpretation of the evidence (Figure 2).

#### 3.5.1. Boxing

Ersoy et al. [32] investigated the influence of boxing on MD, cognitive function, and UL motor function. This activity was performed either through a VR system (Xbox 360 with Kinect) or using real equipment. Both groups completed the same training volume and duration (3 sessions per week for 8 weeks), with 20 participants in each group. Results showed significant improvements in UL motor function, MD, balance, and cognitive function in both groups.

#### 3.5.2. Motor Imagery Training with Brain–Computer Interface (MI-BCI)

Liu et al. [36] evaluated the effects of MI-BCI on UL motor function, ADLs, attention, and visual processing. The sample consisted of 60 participants, equally assigned to the experimental group and the control group, the latter receiving conventional OT. The intervention consisted of 5 sessions of 20 min over 3 weeks. Improvements were observed in both motor and cognitive variables, suggesting a relationship between these domains.

#### 3.5.3. Dual-Task Training

Park et al. [40] studied dual-task training, combining UL motor activities with cognitive tasks related to balance, attention, and memory. The study included 30 participants, divided into an experimental group and a control group (conventional OT), with a frequency of 3 sessions per week for 6 weeks. Results showed significant improvements in cognitive processes, motor performance, and balance, with greater gains in the experimental group.

#### 3.5.4. Cognitive Orientation to Daily Occupational Performance (CO-OP)

Wolf et al. [39] examined the effectiveness of the CO-OP approach to improve attention and executive function, UL function, and DM, quality of life, and occupational performance. Twenty-six adults participated, with up to 10 sessions per participant. The experimental group showed significant improvements in UL function and executive and attentional functions, which were maintained at a 3-month follow-up evaluation.

#### 3.5.5. Robotics and VR

Six studies used robotic and/or VR technologies to improve UL motor function and cognitive performance. Programs ranged from 12 to 48 sessions, lasting 30 to 60 min per session, with sample sizes between 17 and 48 participants. Torrisi et al. [38] and Lee et al. [35] combined robotic and VR rehabilitation, Young-Bi Oh et al. [33] VR combined with real-world objects, Taravati et al. [34] UL robotic rehabilitation program, and the studies of Rogers et al. [31] and Wilson et al. [37] VR-only programs, with Wilson et al. [37] focusing on autonomous home-based rehabilitation. Regarding control groups, four studies used conventional OT; one used computer-based recreational activities [35], and another used the GRASP program [37]. Overall, results demonstrated improvements in cognitive performance (attention, memory, constructive praxis, executive function) and UL function, including MD and fine motor skills. Additional benefits were observed in depression, quality of life, and spasticity.

## 4. Discussion

This scoping review summarized evidence from the last 10 years on UL function, especially MD training, and its relation to cognitive processes in adults with stroke. Following scoping review aims, the synthesis is descriptive, focusing on types of interventions, outcome measures, and methodologies rather than comparing effectiveness.

All 10 studies focused on stroke, likely due to its prevalence and multifactorial impact. The interventions examined encompass diverse approaches such as boxing, MI-BCI, dual-task training, the CO-OP approach, robotics, and VR systems. The interventions examined encompass diverse approaches such as boxing, MI-BCI, dual-task training, the CO-OP approach, robotics, and VR systems, reflecting a broad trend toward task-oriented and cognitively enriched UL rehabilitation. Most studies used robotic and/or VR technologies to improve UL motor function and cognitive performance. All interventions reported beneficial results in UL function, as well as motor and cognitive outcomes. For instance, Ersoy et al. [32] reported comparable cognitive engagement in virtual and real environments, suggesting that the cognitive demands associated with MD tasks may be independent of the delivery medium. This may reflect the demands of fine motor activity, visuomotor processing, planning, and action monitoring, all related to cognition. These results are consistent with the findings reported by Gómez González [41], who observed improvements in attention and executive functions in children with developmental coordination disorder following interventions based on VR and motor imagery. Similarly, Bernate [42] observed associations between motor skills and cognitive outcomes in pediatric and adolescent populations without neurological injury, especially in activities integrating visuomotor coordination and cognitive load. These parallels highlight recurring patterns in which motor and cognitive components co-occur, providing preliminary support for shared motor–cognitive networks and reinforcing the relevance of cognitively enriched MD training across the lifespan.

### 4.1. Clinical Implications for Occupational Therapy

Findings of this review provide an overview of interventions described in the literature relevant to OT. Specifically, the results describe interventions commonly reported in the literature, including task-oriented and cognitively enriched MD training and UL function, with reported motor and cognitive outcomes reflecting task performance, engagement, and functional participation. This work illustrates strategies commonly applied in clinical neurorehabilitation, including MD and UL motor performance, and illustrates patterns that could be considered in planning interventions targeting functional goals, dual-task performance, and independence in ADLs. The dual-task paradigm is frequently reported during UL rehabilitation, integrating fine motor exercises with cognitive demands and highlighting the use of OT approaches within integrated interventions.

Compared with previous systematic reviews, which have primarily focused on technology-based interventions such as VR to address cognitive or motor outcomes in isolation [43,44], or on cognitive strategies such as motor imagery and mental practice mainly targeting motor recovery [45], the present review adopts a more integrative perspective. By specifically examining cognitively enriched motor dual-task interventions, our findings illustrate patterns of integration of cognitive and motor demands during task-oriented training, in line with core OT principles.

### 4.2. Limitations

Despite the encouraging results, certain limitations must be acknowledged. It is not possible to determine whether treatment duration and intensity influence outcomes, as all studies compared groups that received the same number of sessions and intervention time. Future research should explore the optimal dosage of these programs and examine the influence of variables such as injury chronicity, baseline cognitive function, and patient motivation. Additionally, although some studies included follow-up assessments, these were generally short-term; therefore, medium- and long-term evaluations are recommended to determine the persistence of benefits.

It would also be appropriate to increase the etiological diversity of the samples, as the exclusive focus on stroke limits generalizability to other ABI populations, such as traumatic brain injury, brain tumors, or anoxia. Therefore, the observed benefits of integrating MD, UL function, and cognitive components should be interpreted with caution outside the context of stroke. Likewise, standardizing assessment instruments would facilitate comparison across studies and support the development of evidence-based clinical guidelines.

Furthermore, caution is warranted when interpreting the results, given that many of the included studies had relatively small sample sizes. Another limitation of the available evidence is the substantial variability in outcome measures, intervention duration, and assessment procedures across studies. The wide variety of interventions examined and the large number of different tools used to measure outcomes further complicate comparison across studies and limit the generalizability of findings. This heterogeneity limits comparability and should be considered when interpreting the findings or designing future interventions targeting MD and cognitive function. Moreover, this heterogeneity prevented us from reliably determining whether the cognitive improvements observed were clinically significant across interventions. In addition, consistent with the scoping review methodology, this review did not assess effect sizes nor aim to establish causal relationships between interventions and outcomes. These considerations highlight the need for future research to employ larger samples and more standardized methodological and measurement approaches.

Our findings may have been influenced by selection bias, a common limitation in review studies. This bias could have been increased by including only studies published in English or Spanish, which may have led to the omission of relevant studies in other languages. Nevertheless, it is important to note that English is the predominant language of scientific publication, and the majority of research in this field is available in this language. In addition, we used only two databases (PubMed and Scopus). Although they offer broad and complementary coverage of the relevant fields [46], and this database combination has been used in a recently published scoping review [47], the inclusion of additional databases such as CINAHL, Web of Science, or Embase might have identified further studies. Finally, we acknowledge that restricting our review to studies published in the last 10 years and to experimental designs may have also increased selection bias and may not capture earlier foundational work on motor–cognition interactions.

## 5. Conclusions

This scoping review summarizes the available scientific evidence on the relationship between MD training, UL function, and cognitive processes in adults with stroke, based on the outcomes reported in the included studies. The interventions encompassed a wide range of approaches, including boxing, motor imagery with BCI, dual-task training, the CO-OP approach, robotics, and VR. Session durations ranged from 20 to 60 min and the most commonly used assessment tools were the BBT for MD and the MoCA for cognitive function.

All included studies reported outcomes in both motor and cognitive domains, although only one study specifically focused on MD training. Reported outcomes were associated with motor and cognitive performance, particularly in interventions integrating explicit or implicit cognitive components. These findings align with the notion that motor and cognitive processes may occur concurrently within integrated interventions. From an OT perspective, the reviewed studies illustrate commonly reported approaches that combine task-oriented and cognitive elements, highlighting the role of OT within integrated intervention strategies in stroke rehabilitation.

However, future studies should consider increasing sample sizes, standardizing assessment tools, and examining this relationship more directly to obtain more precise and generalizable conclusions.

## Figures and Tables

**Table 1:** Search strategies and databases.

Database	Search Strategy	Results
PubMed		
#1 (P)	“stroke” [Title/Abstract] OR “brain injury” [Title/Abstract]	452,060
#2 (I)	“upper limb” [Title/Abstract] OR “manual training” [Title/Abstract] OR “hand function” [Title/Abstract] OR “manual dexterity” [Title/Abstract] OR “fine motor skills” [Title/Abstract] OR “rehabilitation” [Title/Abstract]	285,929
#3 (C)	NA	
#4 (O)	“cognitive function” [Title/Abstract] OR “executive function” [Title/Abstract] OR “cognition” [Title/Abstract] OR “cognitive” [Title/Abstract] OR “memory” [Title/Abstract]	885,155
	#1 AND #2 AND #4	7159
	#1 AND #2 AND #4 filters: in the last 10 years, Clinical Study	764
Scopus		
#1 (P)	TITLE (stroke OR “brain injury”)	799,568
#2 (I)	TITLE (“upper limb” OR “manual training” OR “hand function” OR “manual dexterity” OR “fine motor skills” OR rehabilitation)	577,844
#3 (C)	NA	
#4 (O)	TITLE (“cognitive function” OR “executive function” OR “cognition” OR “cognitive” OR “memory”)	2,496,911
	#1 AND #2 AND #4	738
	#1 AND #2 AND #4 Filters: in the last 10 years, article and subject area	240

C, comparison; I, intervention; NA, not applicable; O, outcome; P, patient/population.

**Table 2:** Main characteristics of the studies included in this scoping review.

Autor, Year	Design	Sample (*n*), Country	Intervention/ Comparator	Duration Intervention	Study Outcomes
Wolf et al., 2016 [39]	Exploratory RCT	26, EEUU	CO-OP + task-oriented practice/Task-oriented practice alone	10 sessions	The CO-OP + task group showed greater improvements in cognitive function and UL performance.
Oh et al., 2019 [33]	RCT	31, Republic of Korea	Training with real instruments and VR/Conventional OT	30 min/day, 3 days/week, for 6 weeks	EG: showed greater improvements in MMT (shoulder extension), MAS (wrist extension), BBT, 9HPT, and increased pinch strength. EG and CG: showed improvements in MMT (finger extension), FMA-UE, MoCA, and MMSE. Results were maintained for at least 4 weeks after training.
Park et al., 2019 [40]	RCT	30, Republic of Korea	Dual-task training/Conventional OT	6 weeks, 3 sessions/week, total 18 sessions (30 min each per participant)	The dual-task program had a more positive effect than conventional OT on auditory attention, working memory, executive function, and balance.
Rogers et al., 2019 [31]	Pilot	21, Australian	Elements VR system/Conventional OT	4 weeks, 3 sessions/week, 30–40 min per session	Both groups showed improvements in cognitive, functional, and motor outcomes; however, the effects were more pronounced in the EG, particularly in hand motor function (BBT) and cognitive performance. These improvements were maintained at least until the one-month follow-up assessment.
Hye-Sun et al., 2020 [35]	RCT	36, Republic of Korea	Non-immersive VR training with the RAPAEL glove/recreational activity plus conventional OT	30 min/session, 3 days/week, for 8 weeks	Training with a smart glove + non-immersive VR has reasonable and beneficial effects on UL function and cognitive function in chronic stroke survivors.
Ersoy et al., 2021 [32]	RCT	40, Turkey	Real Boxing/Virtual Boxing	24 sessions/3 per week for 8 weeks	Improvements in UL function, balance, and cognitive performance; no significant differences between virtual and real environments. EG: improvement in memory and verbal fluency. CG: improvement in verbal fluency, language, and visuospatial skills.
Torrisi et al., 2021 [38]	RCT	48, Italy	Robotic hand training (Amadeo™) + VR/UL/hand-focused OT without the robotic device + VR	40 sessions/1 h each	EG > greater improvements than CG on the ROCF, MoCA, AM, FAB, and FMA-UE scales. Improvements in working memory, abstract reasoning, and UL motor aspects.
Wilson et al., 2021 [37]	RCT	17, Australian	Virtual home rehabilitation (EDNA 22) plus Conventional rehabilitation/Graded Repetitive Arm Supplementary Program (GRASP) arm training	8 weeks, 30 min per session, 3–4 sessions per week	The EDNA-22 virtual home rehabilitation system demonstrated greater improvements in affected-hand function and moderate improvements in the unaffected hand (BBT/9HPT), as well as moderate gains in cognition (MoCA) and participation/daily living function (SIS/NFI). Improvements were maintained at the 3-month follow-up.
Taravati et al., 2022 [34]	RCT	37, Turkey	Robotic rehabilitation/Conventional OT	30–45-min sessions, 5 days per week, for 4 weeks	Both groups improved in motor function, spasticity reduction, functional independence, quality of life, ADLs, and cognition after 4 weeks. A statistically significant difference between the robotic group and the control group was observed only on the CES-D scale (emotional status/depression) (*p* < 0.05), with the robotic rehabilitation group showing better outcomes.
Liu et al., 2023 [36]	RCT	60, China	MI-BCI training plus conventional rehabilitation/conventional Rehabilitation	3-week intervention, 5 sessions per week, 20 min per MI-BCI session	MI-BCI intervention combined with conventional rehabilitation significantly improved both UL function and attention.

ADLs: Activities of Daily Living; AM: Attentive Matrices; BBT: Box and Block Test; CES-D: Center for Epidemiologic Studies Depression Scale; CG: control group; CO-OP: Cognitive Orientation to daily Occupational Performance; EG: experimental group; FAB: Frontal Assessment Battery; FMA (UE): Fugl-Meyer Assessment (Upper extremity); MAS: Modified Ashworth Scale; MMSE: Mini-Mental State Examination; MMT: Manual Muscle Test; MOCA: Montreal Cognitive Assessment; NFI: Neurobehavioral Functioning Inventory; OT: occupational therapy; RCT: randomized controlled trial; ROCF: Rey–Osterrieth complex figure; SIS: Stroke Impact Scale; UL: upper limb; VR: reality virtual; 9HPT: Nine Hole Peg Test.

## Data Availability

No new data were created or analyzed in this study.

## Supplementary Materials

The following supporting information can be downloaded at https://www.mdpi.com/article/10.3390/healthcare14020234/s1, Table S1: Preferred reporting items for systematic reviews and meta-analyses extension for scoping reviews (PRISMA-ScR) checklist.
